# Cirrhosis and Coagulopathy: Mechanisms of Hemostasis Changes in Liver Failure and Their Management

**DOI:** 10.7759/cureus.23785

**Published:** 2022-04-03

**Authors:** Rabia Islam, Sumana Kundu, Surajkumar B Jha, Ana P Rivera, Gabriela Vanessa Flores Monar, Hamza Islam, Sri Madhurima Puttagunta, Ibrahim Sange

**Affiliations:** 1 Research, Faisalabad Medical University, Faisalabad, PAK; 2 Research, R.G. Kar Medical College, Kolkata, IND; 3 Research, Jinan University School of Medicine, Guangzhou, CHN; 4 Research, Universidad Americana (UAM) Facultad de Medicina, Managua, NIC; 5 Research, Universidad Central del Ecuador, Quito, ECU; 6 Research, Dr Pinnamaneni Siddhartha Institute of Medical Sciences, Chinoutpalli, IND; 7 Research, K. J. Somaiya Medical College, Hospital and Research Center, Mumbai, IND

**Keywords:** hemeostatic system, portal vein thrombosis, thrombocytopenia, direct acting oral anticoagulant, antifibrinolytics, blood coagulation factors, cirrhosis, gastrointestinal hemorrhage, deep vein thrombosis (dvt), venous thromboembolism (vte)

## Abstract

Cirrhosis is an end-stage liver disease that can cause changes in any component of the hemostatic system. The net effects of the complicated hemostatic changes have long been unknown due to concurrent changes in pro-and antihemostatic drivers. Coagulation disorders are caused by various factors, including decreased clotting and inhibitor factor synthesis, reduced clearance of activated factors, quantitative and qualitative platelet defects, hyperfibrinolysis, and increased intravascular coagulation. This review discusses the pathogenesis of coagulopathy and multiple studies related to its clinical presentations. This article also highlights an additional problem in the diagnostic and therapeutic approach to this group of patients: the fact that traditional coagulation tests and transfusional strategies may not be reliable for assessing and managing bleeding or thrombotic risks. Hence, multiple management options have been assessed for bleeding and thrombosis in liver disease.

## Introduction and background

Cirrhosis is thought to be the stage at which fibrotic liver disease becomes irreversible, and fibrosis represents the final pathological pathway in the development of chronic liver disease regardless of etiology [1]. It is recognized by the upregulation of the extracellular matrix (ECM), which obliterates the liver's physiological architecture [2]. Since the liver is the site of synthesis for most procoagulant and anticoagulant factors, it also plays a crucial part in hemostasis. Moreover, it produces thrombopoietin (TPO) and several fibrinolytic proteins [3]. As a result, patients with a decreased liver synthetic capacity are more likely to experience bleeding, particularly gastrointestinal bleeding and coagulopathy caused by liver synthetic dysfunction. They are also at risk of thrombosis, primarily in the splanchnic circulation, particularly when platelet counts are increased by transfusion or drug therapy [4]. In addition, the state of hemostasis is affected by anemia, endotoxemia, portal hypertension, renal failure, and underlying liver disease such as hepatocellular carcinoma [5]. Northup et al. [6] have reported that approximately 0.5% of cirrhotic patients were diagnosed with venous thromboembolism (VTE) for the first time. According to one study, the risk of VTE in cirrhotic patients is twofold higher compared to the risk of pulmonary embolism, which is lower compared to the general population [7].

Bleeding is still a common complication in cirrhotic patients and a frequent cause of ICU admission. According to the literature, the incidence of bleeding in ICU admission ranges from 15% to 61%, of which 17-20% of cirrhotic patients experience a new onset of significant bleeding [8]. Patients with liver diseases have bleeding tendencies with prolonged coagulation screening test results, particularly the prothrombin time/international normalized ratio (PT/INR), the apparent reduction of many liver-derived coagulation factors (e.g., II, V, VII, IX, X, and XI), and a decrease in platelet count, all of which lead to a perceived "hypocoagulable" state [9]. Furthermore, it is becoming increasingly clear that patients with liver disease have hypercoagulable components in their hemostatic status [e.g., increases in von Willebrand factor (vWF), factor VIII, and decreases in anticoagulant proteins] [10,11]. Anticoagulation is beneficial when used therapeutically or prophylactically. Successful anticoagulation is related to a lower rate of decompensation and improved survival. Until now, treatment has consisted of low molecular weight heparins and vitamin K antagonists. According to preliminary data, novel non-vitamin K antagonist oral anticoagulants can be used safely in patients with liver cirrhosis [12]. Coagulopathy in liver cirrhosis is a complicated issue, and current coagulation tests and fresh frozen plasma infusion cannot provide suitable strategies for predicting and preventing bleeding episodes. There is no consensus on the treatment of bleeding complications in decompensated liver cirrhotic patients, and hence more clinical research is needed [13]. The proper management of coagulopathy in decompensated liver cirrhotic patients is a highly debated topic in hepatology. This review article explores the pathologic background for coagulopathy in liver disease and discusses the clinical implications and management.

## Review

Pathogenesis

**Figure 1:**
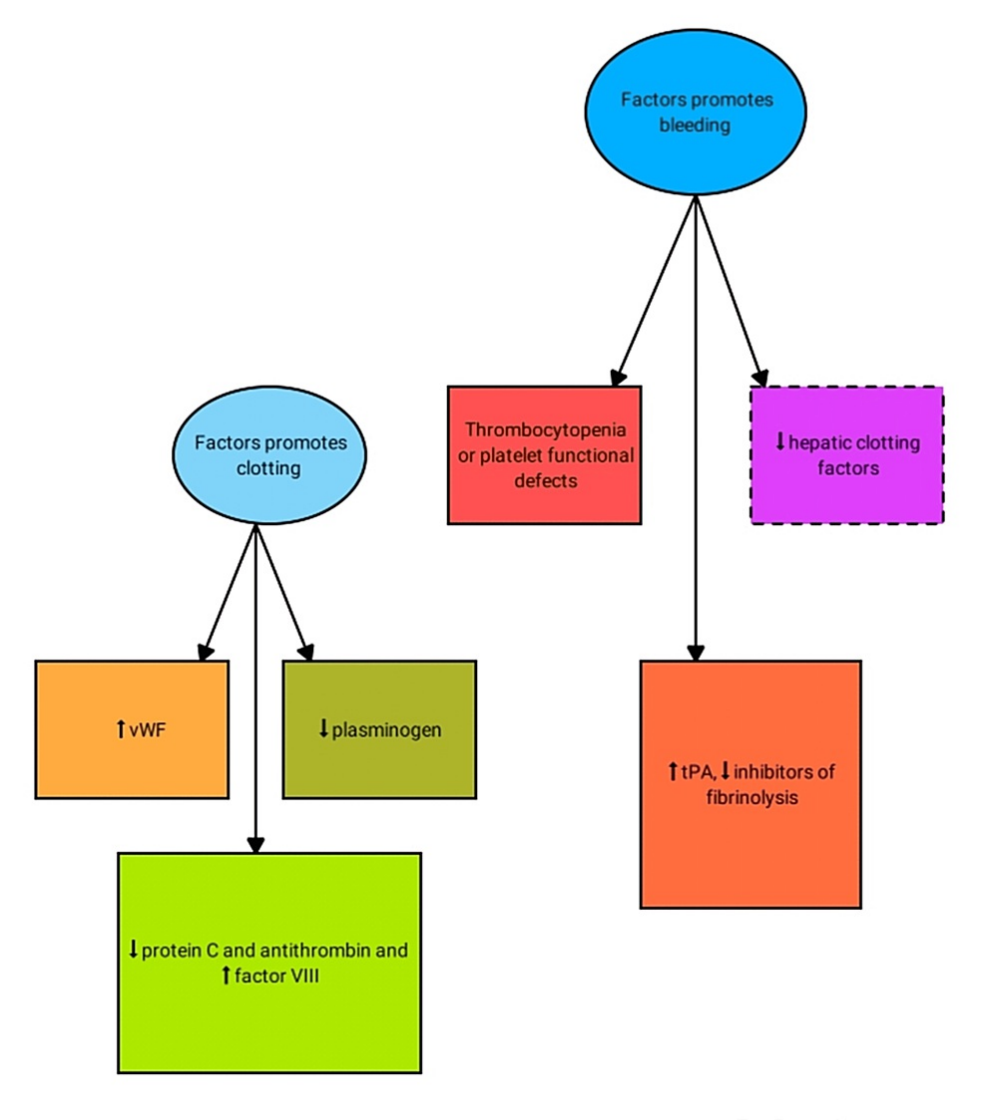
Changes in the coagulation and fibrinolytic system in liver disease vWF: von Willebrand factor; tPA: tissue plasminogen activator

Given the liver's critical role in the coagulation process, liver diseases result in a wide range of hemostasis issues, including quantitative and qualitative platelet abnormalities, a reduced yield of coagulation and inhibitor factors, formulation of abnormal clotting factors, decreased removal of activated factors by the reticuloendothelial system, and hyperfibrinolysis [14]. Hemostasis refers to the formation of blood clots to reduce potential blood loss caused by vessel injury. Platelets bind to the subendothelial matrix via two platelet collagen receptors, strengthening the adhesion by interacting with the vWF. More activated platelets are enrolled and aggregated to form a platelet plug by connecting to fibrinogen molecules via GPIIb/IIIa receptor [15]. For secondary hemostasis, the activated platelet's negative anionic phospholipid surface serves as a substrate via the commencement of the coagulation cascade and subsequent thrombus formation. Factors IX and X are activated by first contacts between TF and factor VII. Eventually, prothrombin (II) transforms to thrombin (IIa) by factor Xa, in collaboration with active factor V. Thrombin transforms fibrinogen to fibrin, and activated factor XIII (found on fibrin) cross-links fibrin, resulting in a complex and stable clot. This mechanism takes place in tandem with regulatory pathways that limit coagulation. Protein C (together with its cofactor protein S) is activated by endothelial-bound thrombomodulin (TM), which degrades Va and VIIIa, preventing further thrombus formation. The fibrinolytic system eliminates fibrin and remodels the thrombus. Tissue plasminogen activator (tPA), plasminogen, alpha-2-antiplasmin, and thrombin-activatable fibrinolysis inhibitor (TAFI) are all critical fibrinolytic system effectors. The bulk of proteins in the coagulation system is produced by hepatocytes. As a result, both acute and chronic liver damage can have a considerable impact on the synthesis of pro-and anticoagulant proteins, as well as platelets (Figure 1) [16].

**Figure 2:**
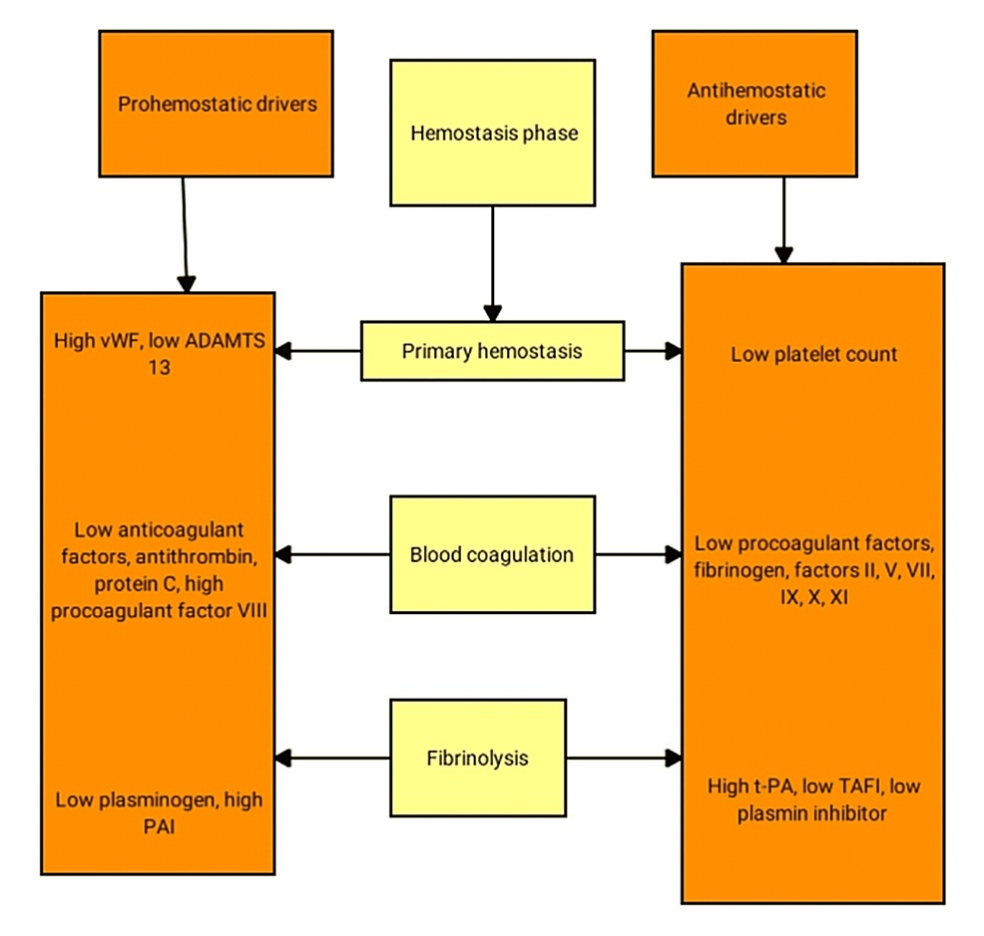
Trends of prohemostatic and antihemostatic drivers in the different phases of hemostasis in chronic liver disease patients ADAMTS 13 denotes disintegrin and metalloprotease with thrombospondin type 1 motif 13 PAI: plasminogen activator inhibitor; TAFI: thrombin-activatable fibrinolysis inhibitor; tPA: tissue plasminogen activator

Prohemostatic and antihemostatic drivers in the different phases of hemostasis in chronic liver disease patients are discussed below (Figure 2).

Defects in primary hemostasis in liver disease 

The main cause of thrombocytopenia in cirrhotic patients is thought to be the increased platelet sequestration in the spleen due to congestive splenomegaly [17]. Decreased production of TPO by the affected liver has been suggested to contribute to the low platelet count in patients with chronic or acute liver failure [18]. Platelet function defects are common in patients with chronic or acute liver disease. Platelet vessel wall interaction may be impaired in patients with liver disease due to proteolysis of platelet receptors by plasmin [19] or the presence of a low hematocrit [20]. Plasma levels of vWF are significantly higher in patients with liver failure, presumably due to endothelial dysfunction caused by endotoxemia [21]. However, cirrhotic patients have lower levels of the vWF cleaving protease, which cleaves high molecular weight vWF multimers into less reactive smaller multimers [22].

Defects in secondary hemostasis in liver

1. Defects in Procoagulant Pathways

Patients with liver failure have dysfunctional proteins and low levels of clotting factors due to impaired synthesis capacity. Factors II, VII, IX, and X, as well as the anticoagulant factors protein C and protein S, contain γ-carboxyglutamic acid (GLA) residues that aid in binding anionic phospholipids. Abnormal γ-carboxylation could be caused by an intrinsic enzymatic defect or a vitamin K deficiency [23]. Factor V, a vitamin K-independent coagulation factor, is also decreased in chronic and acute hepatic failure [24]. Fibrinogen levels in patients with stable chronic liver disease are normal, but decreased levels are found in patients with advanced cirrhosis or acute hepatic failure [25]. Increased clearance and consumption due to intravascular coagulation may also contribute to the hypofibrinogenemia seen in these patients. Abnormal fibrinogen molecules are commonly found in chronic and acute liver failure [25].

2. Defects in Anticoagulant Pathways 

Plasma levels of tissue factor pathway inhibitor (TFPI) in patients with liver diseases tend to be normal, though significantly lower levels have also been reported [26]. Normal levels would be expected because endothelial cells (rather than hepatocytes) are thought to be the primary site of TFPI synthesis [27]. Antithrombin (AT) levels in liver failure are reduced, resulting in decreased thrombin inhibition [28]. Protein C and S levels are lower in patients with liver disease due to reduced synthesis. Furthermore, protein C and S molecules lacking γ-carboxylation could be produced [28]. Low levels of anticoagulant proteins may not only compensate for the impaired thrombin-generating capacity caused by low levels of procoagulant proteins in patients with liver failure but may also facilitate thrombosis [29].

3. Disorders of the Fibrinolytic System 

Except for tPA and plasminogen activator inhibitor (PAI), all proteins involved in fibrinolysis are synthesized in the liver, and patients with liver diseases have lower plasma levels of plasminogen, α2-antiplasmin, factor XIII, and TAFI [30]. Plasma tPA levels are frequently elevated in patients with liver failure due to either increased secretion from endothelial cells or decreased clearance by the diseased liver [31]. PAI levels in plasma from patients with liver disease are slightly elevated and do not appear to balance the high plasma tPA levels, except in acute hepatic failure, where plasma PAI levels are dramatically elevated [32]. The net effect of the changes in the fibrinolytic system in patients with chronic liver failure is thought to be a hyperfibrinolytic state [13]. Figure 2 below illustrates the trends of prohemostatic and antihemostatic drivers in the different phases of hemostasis in chronic liver disease patients.

Clinical manifestations 

For many decades, bleeding was considered a major clinical issue, whereas inadequate clotting is now recognized and associated with alterations in the hemostatic balance [33].

Bleeding can be broadly classified into two types: (1) portal pressure-driven bleeding with little relevance to hemostatic pathways, and (2) mucosal or laceration wound bleeding with a constituent of premature clot dissolution or hyperfibrinolysis, referred to as accelerated intravascular coagulation and fibrinolysis in liver disease [33]. Cirrhotic patients experience spontaneous bleeding, which includes gastrointestinal blood loss and cerebral hemorrhage, although there is no link between coagulation abnormalities and bleeding in both situations [34]. Notably, intracerebral hemorrhage is a rare complication more closely related to the etiology of the disease, such as 0.3% in virus-related cirrhosis and 1.8% in alcoholic cirrhosis, than to the intensity of the liver damage [35]. The limited relationship between clotting alterations and bleeding is also more visible in the case of triggered hemorrhage for two reasons. First, bleeding was uncommon in patients undergoing invasive procedures such as liver biopsy, central venous catheterization, or paracentesis. Second, even in the presence of bleeding, there is no link between clotting and platelet changes and induced bleeding [34]. 

Observational studies in the general population have found that patients with liver diseases have a higher risk of VTE than patients who do not have liver diseases [36]. Furthermore, patients with non-alcoholic steatohepatitis (NASH)-related cirrhosis are at a higher risk of VTE events than patients with other causes of liver diseases [37]. Wu et al. conducted a retrospective study on the relationship between VTE and death rates in a large sample of 649,879 cases; the correlation with VTE appears to be a consequence of the poor outcome, indicating that the risk of mortality in cirrhotic individuals with VTE is higher regardless of the degree of their liver failure. In addition, population-based statistics show that cirrhosis patients have an elevated relative risk of VTE [38]. Søgaard et al. conducted the most extensive study on this issue, assessing the incidence of VTE and pulmonary embolism in 99,444 VTE patients and 496,872 control groups. The study discovered that cirrhotic patients have nearly twice the risk of VTE and a slightly lower risk of pulmonary embolism, and there was no difference when the analysis was restricted to cirrhosis with unprovoked VTE [39]. Gulley et al. conducted a retrospective study to reveal a link between cirrhosis and VTE. However, there was no evidence given for VTE predictor variables, but it was discovered that patients with moderate-severe liver failure had a higher incidence of VTE, though the variation with patients with mild liver failure was not considerable [40]. Northup et al. investigated VTE incidence in over 21,000 cirrhotic patients admitted to a hospital in 2006. They observed that approximately 0.5% of cirrhotic patients had a new diagnosis of VTE, and serum albumin was an accurate indicator of VTE in a multivariate logistic assessment (Table 1) [41]. 

However, portal vein thrombosis (PVT) is the most common thrombotic complication. Its prevalence varies widely: in compensated disease, it ranges between 0.6 and 5%, but in advanced disease, it can reach up to 20-25% [42]. According to the Virchow triad, PVT is caused by hypercoagulation, endothelial damage, and decreased flow velocity [43]. PVT can be fatal as it is linked to vascular outcomes that can dramatically deteriorate the clinical picture of cirrhosis. Gastrointestinal blood loss due to a rise in blood pressure in portal circulation is one of the most frequent complications [42]. Intestinal infarction caused by a thrombus extending from the portal vein to the mesenteric vein can be fatal if not recognized instantly [44]. Table 1 summarizes the studies related to the incidence of thrombotic events in cirrhotic patients.

**Table 1:** Studies related to the incidence of thrombotic events in cirrhotic patients NR: not reported; VTE: venous thromboembolism; DVT: deep vein thrombosis; PE: pulmonary embolism; INR: international normalized ratio

Author/year	Study design	Subject population	Sample size	Study period	Conclusion	Comments
Wu et al. (2010) [38]	NR	Discharged patient with cirrhosis	649,879 patients	1998-2006	VTE was associated with increased mortality in patients with cirrhosis	Cirrhotic patients with age <45 years are at higher risk of VTE than those without liver disease and VTE prophylaxis should be considered
Sogaard et al. (2009) [39]	Case-control study	Patient with liver disease with no underlying cause of VTE	99,444 patients, 49,6872 control	1980-2005	Patients with liver disease have an increased risk of VTE	The risk of DVT was higher than PE
Gulley et al. (2008) [40]	Case-control study	Hospitalized patients with cirrhosis	963 patients, 12,405 control	NR	Both incidences of DVT/PE and comorbidity were higher in cirrhotics	Partial thromboplastin time and serum albumin were independent predictors of DVT/PE in cirrhotics
Northup et al. (2006) [41]	Retrospective case-control study	Hospitalized patients with cirrhosis	113 patients	Eight-year period	Approximately 0.5% of admissions of cirrhotic patients result in a new thrombotic event	Low serum albumin was a strong predictor of increased risk of VTE independent of INR or platelet

Investigative parameters

In clinical settings, reliance on conventional coagulation tests such as the PT, INR, and activated partial thromboplastin time (aPTT) is routine to assess bleeding risk, evaluate unexplained bleeding, and monitor the effect of anticoagulants such as heparin and warfarin [16]. Although these tests are routinely abnormal in cirrhotic patients, they do not accurately measure bleeding or clotting risk, and relying on them alone can be misleading. The PT/INR, for example, assesses only procoagulant features of the coagulation system and does not assess counterbalancing anticoagulant factors, such as protein C, which interact in vivo to inhibit clot formation. Protein C, an essential regulator of clot formation, is significantly decreased in cirrhosis, and when activated, it degrades factors Va and VIIIa [16].

The other pillar of conventional laboratory investigations is platelet count and fibrinogen level. Because of the interconnection of retrospective and in vitro data suggesting that platelet counts in the 50,000-60,000 range enhance thrombin output, this range has been designated as the target for prophylactic treatment [45]. However, in cirrhosis, increased vWF and elevated circulating activated platelets are mitigating variables that have not been considered [46]. Single-donor platelet transfusion reduces immunologic risk when enhanced platelet levels are judged critically. Cirrhosis-related thrombocytopenia is complex, involving decreased platelet survival, platelet sequestration, and insufficient bone marrow response. Consequently, when treatments are scheduled ahead of time, newly licensed TPO agonists are an excellent substitute for platelet transfusion [47]. Fibrinogen levels have emerged recently as potentially more appropriate than INR as an indicator of bleeding risk when combined with platelet levels [8].

Alternative coagulation tests such as vasoelastic test and thrombin generation assay (TGA) in cirrhosis have been described as potentially more accurate. Despite the field's rapid advancement, TGA has been critical in increasing our understanding of hemostatic mechanisms in cirrhosis. However, it appears unlikely to be translated into a practical treatment modality. Whole blood tests [including thromboelastography (TEG), rotational thromboelastometry (ROTEM), and sonorheometry] are referred to as "viscoelastic elastic tests," and they may be an excellent way to imitate the in vivo studies of the hemostatic pathways [48]. These diagnostics rely on changes in resistance to motion or ultrasonic density measures to determine the thickness of a developing clot [48].

Ultrasound can diagnose the development of portal hypertension by assessing portal vein diameter, flow velocity, ascites, and splenomegaly [49]. Multiphase CT is another option for diagnosing PVT during cirrhosis diagnosis. Ultrasound can identify thrombus in the portal vein trunk and intrahepatic branches. CT allows for a more accurate assessment of the superior mesenteric vein, the existence of portosystemic shunts, renal veins, and the inferior vena cava, as well as the degree of thrombus. CT scans can aid in diagnosing hepatocellular carcinoma and intestinal ischemia [50]. MRI with contrast, like CT, helps reveal portal venous system flow and thrombus [51].

Prevention and management of bleeding in cirrhosis

Patients with a platelet count above 50,000/l are less likely to experience acute bleeding or bleeding during minor invasive procedures [52]. Platelet transfusion is recommended during bleeding events or prior to invasive procedures (e.g., liver biopsy) when platelet counts are less than 50,000/l [53]. It is debatable if a low preprocedural platelet count increases the likelihood of procedural hemorrhage [45]. Giannini et al. recorded invasive procedures and incidences of procedure-related bleeding among 121 patients and noted that bleeding related to invasive procedures occurs most frequently in patients with severe thrombocytopenia, whereas significant coagulopathy does not seem to be associated with bleeding [45]. Despite this, preprocedural platelet concentrate administration is still popular, and TPO binding site agonists are regarded as possible alternative options to platelet transfusions [54]. They may be linked with an increased risk of thromboembolic events [55]. Afdhal et al. reported that, as compared with placebo, the administration of eltrombopag, an oral TPO-receptor agonist, for 14 days in patients with cirrhosis and thrombocytopenia increased the platelet count and reduced the need for platelet transfusion but was associated with an increased risk of PVT [56]. The prolonged bleeding time in patients with liver failure can be corrected by infusion of 1-deamino-8-d-arginine vasopressin (DDAVP), but the actual mechanism and clinical significance of this finding are unknown [57].

Antifibrinolytics have been shown to decrease blood components consumption after liver transplant and given their relative safety, they may be useful in a preventive setting [58]. Porte et al. conducted a randomized, double-blind, placebo-controlled trial in which 137 patients undergoing primary liver transplantation were randomly assigned intraoperative high-dose aprotinin (antifibrinolytic), regular-dose aprotinin, or placebo. They concluded that intraoperative use of aprotinin markedly reduces blood-transfusion requirements and should be routinely used in patients without contraindications [58].

Theoretically, prophylactic coagulation correction with low-volume products such as prothrombin complex concentrates outperforms plasma administration [59]. Finally, an organized bleeding history should be included in the workup of patients with liver disorders for (elective) invasive procedures. When blood loss occurs, determining the most beneficial prohemostatic therapy can be challenging because bleeding can be caused by any of the hemostatic systems failing. Thromboelastography may help determine which factors need to be corrected. Although thromboelastography-based transfusion algorithms are rapidly gaining popularity, there is a lack of convincing clinical evidence that such algorithms result in optimal hemostatic capacity restoration [60].

Prevention and management of thrombosis in cirrhosis

VTE in cirrhosis is a growing clinical problem, demonstrating once again that patients with liver cirrhosis are not naturally protected from thrombotic events. The low platelet count and elevated INR levels in patients with liver failure do not reduce the risk of developing deep vein thrombosis or pulmonary embolism [61]. Prophylactic anticoagulation is well established and suggested in guidelines for the general population of inpatients. Still, there are significant concerns about using anticoagulation prophylactically in patients with cirrhosis for fear of facilitating bleeding events [62].

The most commonly used agents are heparins [e.g., unfractionated heparin, low-molecular-weight heparin (LMWH), fondaparinux] and vitamin K antagonists (VKAs) such as warfarin. The most widely investigated therapeutics in cirrhotic patients are LMWH and VKA. Direct-acting oral anticoagulants (DOACs) that directly inhibit factor Xa (apixaban, rivaroxaban, and edoxaban) or factor IIa (dabigatran) are increasingly being used in patients for a wide range of conditions [63].

LMWH (enoxaparin, for example) inhibits factor Xa by binding to AT. Its advantages in cirrhotic patients include documented safety and efficacy and the possibility to use it in an outpatient clinic without routine testing [64]. Bechmann et al. studied anti-Xa levels in patients with cirrhosis receiving LMWH and concluded that the standard doses failed to reach the recommended anti-Xa levels and implied that LMWH doses should be increased in those patients not achieving the desired anti-Xa target range. "Overanticoagulation" of patients with cirrhosis would likely result in an increased bleeding risk, with potentially devastating consequences [65]. The patient's discomfort with repeated subcutaneous injections is a clear disadvantage of LMWH, which leads to non-compliance. Another possible contraindication for LMWH use is renal impairment, common in cirrhosis. As a result of the lower financial implications and the more desirable oral method of delivery, clinicians commonly select VKA for long-term treatment. Vitamin K-dependent procoagulant proteins II, VII, IX, and X, along with anticoagulant proteins C and S, are all inhibited by VKA. VKA has several benefits, including minimal cost, ease of administration, and the capability to counter the effect with vitamin K and exogenous coagulation factor replacement. On the other hand, VKAs require regular monitoring and adjustment with an INR, as well as diet compliance, and have a very limited therapeutic window. VKAs are inappropriate for cirrhotic patients, who often have increased INR due to the lack of coagulation factor synthesis because they depend on INR for monitoring. Several studies have demonstrated that INR does not anticipate the risk of bleeding or clotting in cirrhosis, and interlaboratory fluctuation is common [66,67].

DOACs are gaining popularity as a treatment and prophylaxis for thrombosis. Direct factor Xa inhibitors (apixaban, rivaroxaban, and edoxaban) and direct factor IIa inhibitors (dabigatran) are available in the United States. DOACs are not recommended in decompensated cirrhosis. A recent report suggests that rivaroxaban may cause direct hepatic injury apart from the underlying problems of dosing, efficacy, and bleeding risk [68]. Russman et al. experimented with absolute and relative risks of rivaroxaban on 14 cirrhotic patients and concluded that if no other cause of hepatic injury had been excluded, then rivaroxaban should be stopped immediately [68]. On the other hand, an extensive systematic review and meta-analysis of 29 phase-III randomized controlled trials (RCTs) conducted by Calderia et al. concluded that DOACs are not associated with an increased risk of drug-induced liver injury. The unexpected "protective" effect of DOAC is probably due to LMWH-associated hepatotoxicity [69]. The advantages of DOAC compared to conventional therapy are its ease of administration without monitoring, which is desired by patients and clinicians [70]. DOACs' disadvantages include high cost, limited safety data in cirrhotic patients, and a perceived lack of a "reversal agent" [71].

There are several management options for patients with PVT, including anticoagulation, transjugular intrahepatic portosystemic stent-shunt (TIPSS), and endovascular procedures with fibrinolysis. Patients at risk of developing PVT may also benefit from primary preventative strategies. Wang et al. conducted a randomized controlled study in 2016 to see if post-transjugular intrahepatic portosystemic shunt (TIPS) placement anticoagulation therapy on 31 patients could benefit patients with cirrhosis and PVT in terms of portal vein patency status and clinical outcomes, and they concluded that anticoagulation therapy might not be necessary for certain patients with PVT because TIPS placement alone can achieve a high persistent recanalization rate [72]. Another cross-sectional study performed by Chen et al. in 2015 regarding the effect of anticoagulation in 30 cirrhotic patients with coagulopathy concluded that anticoagulation with warfarin might result in the resolution of more advanced PVT effectively and safely in patients with liver cirrhosis. In addition, there was no evidence of the benefit of anticoagulation for decompensation or death [73]. Chung et al. conducted a retrospective cross-sectional study on 14 cirrhotic patients with nonmalignant PVT treated with warfarin and concluded that warfarin could be safely given to cirrhotic patients with nonmalignant PVT and the presence of preexisting portal hypertension is a predictor of nonresponse to anticoagulation [74]. Senzolo et al. conducted a cross-sectional study in 2012 that aimed to evaluate anticoagulation and TIPS to treat PVT in 35 cirrhotic patients. They concluded that LMWH offers a good chance of complete repermeation, reduces portal hypertensive complications, and reduces the rate of thrombosis progression (Table 2) [75].

**Table 2:** Summary of studies reporting the use of anticoagulation for portal vein thrombosis in cirrhosis *Two patients were excluded NR: not reported; P: prospective; PVT: portal vein thrombosis; R: retrospective; RCT: randomized control study; LMWH: low-molecular-weight heparin; CS: cross-sectional study

Author/year	Study design	Study population	Anticoagulated patients/controls	Age (years)	Follow-up (months)	Duration of anticoagulation (months)	Type of anticoagulation	PVT recanalization	PVT unchanged	PVT extension	Bleeding outcomes
Wang et al. (2016) [72]	P, RCT	Cirrhotic patients with PVT who underwent TIPS placement	31 treated	54.5	12	12	Warfarin	31/31	0/31	0/31	3 gastrointestinal (1 variceal)
			33 untreated	55				30/32	1/32	1/32	2 gastrointestinal (1 variceal)
Chen et al. (2015) [73]	R, CS	Cirrhotic patients with nonmalignant PVT	30 treated	44.9	33	7.6	Warfarin	15/22	4/22	3/22	4 hematemesis/malena, 1 epistaxis, 3 gingival
			36 untreated	47.8				4/16	6/16	6/16	NR
Chung et al. (2014) [74]	R, CS	Cirrhotic patients with nonmalignant PVT	14 treated	59.4	4	3.7	Warfarin	11/14 (6 complete, 5 partial)	2/14	1/14	NR
			14 untreated	58.7				5/14 (3 complete, 2 partial)	2/14	3/14	1 variceal, 1 subarachnoid hemorrhage
Senzolo et al. (2012) [75]	P, CS	Cirrhotic patients with nonmalignant PVT	35 treated*	55.5	24	6	LMWH	12/33 complete, 9/33 partial (>50%)	7/33	5/33	1 cerebral, 1 epistaxis, 1 hematuria, 1 variceal
			21 untreated	52.3				1/21	NR	15/21	5 variceal

Limitations

The coagulopathy that has been reviewed in this article is related to cirrhosis only. In contrast, the etiology of coagulopathy depends on different factors that are not discussed in this article; for example, multifactorial causes of deep vein thrombosis include pregnancy, oral contraceptive pills, trauma, and hip surgeries. Therefore, all relevant data related to coagulopathy was not evaluated.

## Conclusions

Cirrhosis is a medical entity associated with a high risk of systemic thromboembolism and bleeding. Patients may present with both bleeding and thrombosis, and treating such patients is a complex clinical challenge. Treatment options for the majority of patients with cirrhosis and VTE include heparin, VKAs such as warfarin, and DOACs. Anticoagulants are most effective in PVT prophylaxis and management. Platelet transfusion, TPO receptor agonist, and antifibrinolytics are beneficial in bleeding complications of cirrhosis. We believe this article can help clinicians overcome the challenges by taking a comprehensive view of the correlation between the two elements, i.e., cirrhosis and coagulopathy, and highlighting the pathogenesis, investigatory measures, and management options. Keeping good anticoagulation quality and reducing modifiable factors for bleeding can boost success rates and enhance the effectiveness and safety of patients. Finally, we strongly feel that the link between cirrhosis and coagulation requires further deep-insight research studies to develop a more structured and direct approach to diagnosing, managing, and preventing these conditions. However, up till now, the role of anticoagulants seems integral in the prevention and management of VTE in cirrhosis.

## References

[1] Arthur MJ, Iredale JP (1994). Hepatic lipocytes, TIMP-1 and liver fibrosis. J R Coll Physicians Lond.

[2] Iredale JP (2007). Models of liver fibrosis: exploring the dynamic nature of inflammation and repair in a solid organ. J Clin Invest.

[3] Lisman T, Leebeek FW, de Groot PG (2002). Haemostatic abnormalities in patients with liver disease. J Hepatol.

[4] Violi F, Basili S, Raparelli V, Chowdary P, Gatt A, Burroughs AK (2011). Patients with liver cirrhosis suffer from primary haemostatic defects? Fact or fiction?. J Hepatol.

[5] Blasi A (2015). Coagulopathy in liver disease: lack of an assessment tool. World J Gastroenterol.

[6] Ambrosino P, Tarantino L, Di Minno G (2017). The risk of venous thromboembolism in patients with cirrhosis. A systematic review and meta-analysis. Thromb Haemost.

[7] Søgaard KK, Horváth-Puhó E, Montomoli J, Vilstrup H, Sørensen HT (2015). Cirrhosis is associated with an increased 30-day mortality after venous thromboembolism. Clin Transl Gastroenterol.

[8] Drolz A, Horvatits T, Roedl K (2016). Coagulation parameters and major bleeding in critically ill patients with cirrhosis. Hepatology.

[9] Lisman T, Porte RJ (2010). Rebalanced hemostasis in patients with liver disease: evidence and clinical consequences. Blood.

[10] Tripodi A, Primignani M, Mannucci PM (2010). Abnormalities of hemostasis and bleeding in chronic liver disease: the paradigm is challenged. Intern Emerg Med.

[11] Groeneveld D, Porte RJ, Lisman T (2014). Thrombomodulin-modified thrombin generation testing detects a hypercoagulable state in patients with cirrhosis regardless of the exact experimental conditions. Thromb Res.

[12] Turco L, de Raucourt E, Valla DC, Villa E (2019). Anticoagulation in the cirrhotic patient. JHEP Rep.

[13] Caldwell SH, Hoffman M, Lisman T (2006). Coagulation disorders and hemostasis in liver disease: pathophysiology and critical assessment of current management. Hepatology.

[14] Páramo JA, Rocha E (1993). Hemostasis in advanced liver disease. Semin Thromb Hemost.

[15] Savage B, Shattil SJ, Ruggeri ZM (1992). Modulation of platelet function through adhesion receptors. A dual role for glycoprotein IIb-IIIa (integrin alpha IIb beta 3) mediated by fibrinogen and glycoprotein Ib-von Willebrand factor. J Biol Chem.

[16] Intagliata NM, Davis JP, Caldwell SH (2018). Coagulation pathways, hemostasis, and thrombosis in liver failure. Semin Respir Crit Care Med.

[17] Aster RH (1966). Pooling of platelets in the spleen: role in the pathogenesis of "hypersplenic" thrombocytopenia. J Clin Invest.

[18] Goulis J, Chau TN, Jordan S, Mehta AB, Watkinson A, Rolles K, Burroughs AK (1999). Thrombopoietin concentrations are low in patients with cirrhosis and thrombocytopenia and are restored after orthotopic liver transplantation. Gut.

[19] Pasche B, Ouimet H, Francis S, Loscalzo J (1994). Structural changes in platelet glycoprotein IIb/IIIa by plasmin: determinants and functional consequences. Blood.

[20] Turitto VT, Baumgartner HR (1975). Platelet interaction with subendothelium in a perfusion system: physical role of red blood cells. Microvasc Res.

[21] Ferro D, Quintarelli C, Lattuada A, Leo R, Alessandroni M, Mannucci PM, Violi F (1996). High plasma levels of von Willebrand factor as a marker of endothelial perturbation in cirrhosis: relationship to endotoxemia. Hepatology.

[22] Mannucci PM, Canciani MT, Forza I, Lussana F, Lattuada A, Rossi E (2001). Changes in health and disease of the metalloprotease that cleaves von Willebrand factor. Blood.

[23] Wu SM, Morris DP, Stafford DW (1991). Identification and purification to near homogeneity of the vitamin K-dependent carboxylase. Proc Natl Acad Sci U S A.

[24] Bernuau J, Goudeau A, Poynard T (1986). Multivariate analysis of prognostic factors in fulminant hepatitis B. Hepatology.

[25] Francis JL, Armstrong DJ (1982). Acquired dysfibrinogenaemia in liver disease. J Clin Pathol.

[26] Bajaj MS, Rana SV, Wysolmerski RB, Bajaj SP (1987). Inhibitor of the factor VIIa-tissue factor complex is reduced in patients with disseminated intravascular coagulation but not in patients with severe hepatocellular disease. J Clin Invest.

[27] Bajaj MS, Kuppuswamy MN, Saito H, Spitzer SG, Bajaj SP (1990). Cultured normal human hepatocytes do not synthesize lipoprotein-associated coagulation inhibitor: evidence that endothelium is the principal site of its synthesis. Proc Natl Acad Sci U S A.

[28] Lechner K, Niessner H, Thaler E (1977). Coagulation abnormalities in liver disease. Semin Thromb Hemost.

[29] Kemkes-Matthes B, Matthes KJ (1995). Protein Z, a new haemostatic factor, in liver diseases. Haemostasis.

[30] Lisman T, Leebeek FW, Mosnier LO (2001). Thrombin-activatable fibrinolysis inhibitor deficiency in cirrhosis is not associated with increased plasma fibrinolysis. Gastroenterology.

[31] Leiper K, Croll A, Booth NA, Moore NR, Sinclair T, Bennett B (1994). Tissue plasminogen activator, plasminogen activator inhibitors, and activator-inhibitor complex in liver disease. J Clin Pathol.

[32] Pernambuco JR, Langley PG, Hughes RD, Izumi S, Williams R (1993). Activation of the fibrinolytic system in patients with fulminant liver failure. Hepatology.

[33] Joist JH (1999). AICF and DIC in liver cirrhosis: expressions of a hypercoagulable state. Am J Gastroenterol.

[34] Ferro D, Angelico F, Caldwell SH, Violi F (2012). Bleeding and thrombosis in cirrhotic patients: what really matters?. Dig Liver Dis.

[35] Huang HH, Lin HH, Shih YL (2008). Spontaneous intracranial hemorrhage in cirrhotic patients. Clin Neurol Neurosurg.

[36] Mahfood Haddad T, Hamdeh S, Kanmanthareddy A, Alla VM (2017). Nonalcoholic fatty liver disease and the risk of clinical cardiovascular events: a systematic review and meta-analysis. Diabetes Metab Syndr.

[37] Stine JG, Niccum BA, Zimmet AN, Intagliata N, Caldwell SH, Argo CK, Northup PG (2018). Increased risk of venous thromboembolism in hospitalized patients with cirrhosis due to non-alcoholic steatohepatitis. Clin Transl Gastroenterol.

[38] Wu H, Nguyen GC (2010). Liver cirrhosis is associated with venous thromboembolism among hospitalized patients in a nationwide US study. Clin Gastroenterol Hepatol.

[39] Søgaard KK, Horváth-Puhó E, Grønbaek H, Jepsen P, Vilstrup H, Sørensen HT (2009). Risk of venous thromboembolism in patients with liver disease: a nationwide population-based case-control study. Am J Gastroenterol.

[40] Gulley D, Teal E, Suvannasankha A, Chalasani N, Liangpunsakul S (2008). Deep vein thrombosis and pulmonary embolism in cirrhosis patients. Dig Dis Sci.

[41] Northup PG, McMahon MM, Ruhl AP, Altschuler SE, Volk-Bednarz A, Caldwell SH, Berg CL (2006). Coagulopathy does not fully protect hospitalized cirrhosis patients from peripheral venous thromboembolism. Am J Gastroenterol.

[42] Amitrano L, Guardascione MA, Brancaccio V (2004). Risk factors and clinical presentation of portal vein thrombosis in patients with liver cirrhosis. J Hepatol.

[43] Violi F, Ferro D, Basili S (1997). Ongoing prothrombotic state in the portal circulation of cirrhotic patients. Thromb Haemost.

[44] Tsochatzis EA, Senzolo M, Germani G, Gatt A, Burroughs AK (2010). Systematic review: portal vein thrombosis in cirrhosis. Aliment Pharmacol Ther.

[45] Giannini EG, Greco A, Marenco S, Andorno E, Valente U, Savarino V (2010). Incidence of bleeding following invasive procedures in patients with thrombocytopenia and advanced liver disease. Clin Gastroenterol Hepatol.

[46] Raparelli V, Basili S, Carnevale R (2017). Low-grade endotoxemia and platelet activation in cirrhosis. Hepatology.

[47] Terrault NA, Hassanein T, Howell CD (2014). Phase II study of avatrombopag in thrombocytopenic patients with cirrhosis undergoing an elective procedure. J Hepatol.

[48] Davis JP, Northup PG, Caldwell SH, Intagliata NM (2018). Viscoelastic testing in liver disease. Ann Hepatol.

[49] Aubé C, Oberti F, Korali N (1999). Ultrasonographic diagnosis of hepatic fibrosis or cirrhosis. J Hepatol.

[50] Lee HK, Park SJ, Yi BH, Yeon EK, Kim JH, Hong HS (2008). Portal vein thrombosis: CT features. Abdom Imaging.

[51] Wallner B, Edelman RR, Finn JP, Mattle HP (1990). Bright pleural effusion and ascites on gradient-echo MR images: a potential source of confusion in vascular MR studies. AJR Am J Roentgenol.

[52] Sharma P, McDonald GB, Banaji M (1982). The risk of bleeding after percutaneous liver biopsy: relation to platelet count. J Clin Gastroenterol.

[53] Janssen HL (2000). Changing perspectives in portal vein thrombosis. Scand J Gastroenterol Suppl.

[54] Qureshi K, Patel S, Meillier A (2016). The use of thrombopoietin receptor agonists for correction of thrombocytopenia prior to elective procedures in chronic liver diseases: review of current evidence. Int J Hepatol.

[55] Lisman T, Porte RJ (2012). Eltrombopag before procedures in patients with cirrhosis and thrombocytopenia. N Engl J Med.

[56] Afdhal NH, Giannini EG, Tayyab G (2012). Eltrombopag before procedures in patients with cirrhosis and thrombocytopenia. N Engl J Med.

[57] Burroughs AK, Matthews K, Qadiri M, Thomas N, Kernoff P, Tuddenham E, McIntyre N (1985). Desmopressin and bleeding time in patients with cirrhosis. Br Med J (Clin Res Ed).

[58] Porte RJ, Molenaar IQ, Begliomini B (2000). Aprotinin and transfusion requirements in orthotopic liver transplantation: a multicentre randomised double-blind study. EMSALT Study Group. Lancet.

[59] Arshad F, Ickx B, van Beem RT (2013). Prothrombin complex concentrate in the reduction of blood loss during orthotopic liver transplantation: PROTON-trial. BMC Surg.

[60] Wang SC, Shieh JF, Chang KY (2010). Thromboelastography-guided transfusion decreases intraoperative blood transfusion during orthotopic liver transplantation: randomized clinical trial. Transplant Proc.

[61] Dabbagh O, Oza A, Prakash S, Sunna R, Saettele TM (2010). Coagulopathy does not protect against venous thromboembolism in hospitalized patients with chronic liver disease. Chest.

[62] Qaseem A, Chou R, Humphrey LL, Starkey M, Shekelle P (2011). Venous thromboembolism prophylaxis in hospitalized patients: a clinical practice guideline from the American College of Physicians. Ann Intern Med.

[63] Martinez M, Tandra A, Vuppalanchi R (2014). Treatment of acute portal vein thrombosis by nontraditional anticoagulation. Hepatology.

[64] Lisman T, Porte RJ (2011). Towards a rational use of low-molecular-weight heparin in patients with cirrhosis. Liver Int.

[65] Bechmann LP, Sichau M, Wichert M, Gerken G, Kröger K, Hilgard P (2011). Low-molecular-weight heparin in patients with advanced cirrhosis. Liver Int.

[66] Ewe K, Reinhardt P, Müller H, Ohler W (1978). The bleeding time after liver biopsy does not correlate with peripheral coagulation factors. Inn Med.

[67] Trotter JF, Olson J, Lefkowitz J, Smith AD, Arjal R, Kenison J (2007). Changes in international normalized ratio (INR) and model for endstage liver disease (MELD) based on selection of clinical laboratory. Am J Transplant.

[68] Russmann S, Niedrig DF, Budmiger M, Schmidt C, Stieger B, Hürlimann S, Kullak-Ublick GA (2014). Rivaroxaban postmarketing risk of liver injury. J Hepatol.

[69] Caldeira D, Barra M, Santos AT, de Abreu D, Pinto FJ, Ferreira JJ, Costa J (2014). Risk of drug-induced liver injury with the new oral anticoagulants: systematic review and meta-analysis. Heart.

[70] Prins MH, Bamber L, Cano SJ, Wang MY, Erkens P, Bauersachs R, Lensing AW (2015). Patient-reported treatment satisfaction with oral rivaroxaban versus standard therapy in the treatment of pulmonary embolism; results from the EINSTEIN PE trial. Thromb Res.

[71] Lisman T, Kamphuisen PW, Northup PG, Porte RJ (2013). Established and new-generation antithrombotic drugs in patients with cirrhosis - possibilities and caveats. J Hepatol.

[72] Wang Z, Jiang MS, Zhang HL, Weng NN, Luo XF, Li X, Yang L (2016). Is post-TIPS anticoagulation therapy necessary in patients with cirrhosis and portal vein thrombosis? A randomized controlled trial. Radiology.

[73] Chen H, Liu L, Qi X, He C, Wu F, Fan D, Han G (2016). Efficacy and safety of anticoagulation in more advanced portal vein thrombosis in patients with liver cirrhosis. Eur J Gastroenterol Hepatol.

[74] Chung JW, Kim GH, Lee JH, Ok KS, Jang ES, Jeong SH, Kim JW (2014). Safety, efficacy, and response predictors of anticoagulation for the treatment of nonmalignant portal-vein thrombosis in patients with cirrhosis: a propensity score matching analysis. Clin Mol Hepatol.

[75] Senzolo M, M Sartori T, Rossetto V (2012). Prospective evaluation of anticoagulation and transjugular intrahepatic portosystemic shunt for the management of portal vein thrombosis in cirrhosis. Liver Int.

